# Disengaged or raising voices? An analysis of the relationship between individual risk perception and non-institutionalised political participation

**DOI:** 10.1057/s41269-023-00301-x

**Published:** 2023-05-13

**Authors:** Julia Weiss

**Affiliations:** GESIS - Leibniz Institute for the Social Sciences, B6, 4-5, 68159 Mannheim, Germany

**Keywords:** Risk perception, Political participation, Status threat, Economic crisis, Multi-level modelling

## Abstract

The economic crisis of 2008 resulted in rising levels of unemployment, which were accompanied by protests in many countries. This led to a scientific debate on the influence of grievances on political participation. Previous studies have shown that individual affectedness leads to ‘exiting’, whereas in times of crisis grievances lead to increased political participation. What all these studies have in common is that they solely investigate actual deprivation. The present study adds a new aspect and examines if the individual risk perception of becoming deprived influences political participation. Does the individual risk perception of becoming deprived influence political participation and, if so, does this depend on the economic context? Based on grievance theory and using data from the European Social Survey and the World Bank, this study shows that the personal risk of becoming deprived increases the likelihood of participation. However, the participation-enhancing effect of one’s own risk perception is diminished when individuals live in an economically weak country and thus see no chance of improvement. This underlines that context plays a role and that economically deteriorating situations in the country can reduce participation rates.

**Supplementary Information:**

The online version contains supplementary material available at 10.1057/s41269-023-00301-x.

## Introduction

The economic crisis that unfolded in 2008 resulted in negative economic growth, weak economic prospects for many European countries and high levels of unemployment. This economic downturn sparked protests in numerous countries, especially in those hit particularly hard by the recession, as people ‘raised their voices’ (Kriesi 2012) to express their dissatisfaction. In this regard, personal dissatisfaction stimulated political participation.

Although participation in protests has become a more normal form of expressing opinions and political preferences over the decades (Van Aelst and Walgrave 2001), existing research clearly shows that not all social classes in a society participate equally. Whereas in earlier decades political protests were mainly associated with the labour movement and the mobilization of the working class, today mainly well-educated members of the middle class participate (Hylmö and Wennerhag 2015). At the same time, a look at those who nowadays do *not* participate shows that they are often materially deprived, lacking the resources for participation or simply needing their time to make ends meet. In times of crisis, when the number of people affected by economic deprivation increases, the participation of certain social groups is likely to decrease. Simultaneously, the risk of becoming economically deprived also increases for more people in times of crisis. However, it is still unclear how this affects the participation behaviour of this group.

Against this backdrop, this article sets out to analyse the relationship between the individual risk of becoming deprived and reactions to this risk in the form of political participation behaviour, as well as the effect of the economic crisis on this. Several studies on how economic performance affects levels of political participation have focused on actual deprivation. Additionally, other scholars have shown that economic performance affects political participation differently in times of crisis compared to times of non-crisis. This article engages in the ongoing debate on the influence of deprivation on political participation behaviour (Filetti and Janmaat 2018; Grasso and Giugni 2019; Kern et al. 2015; Kurer et al. 2019) by addressing the following question: Does the *individual risk perception of becoming deprived* influence political participation and, if so, does this depend on the economic context?

Despite the fact that much is already known about the role of actual economic deprivation on an individual’s participation behaviour, this analysis will provide new insights into the relationship between economic deprivation and participation behaviour considering those who are not yet deprived but perceive the risk of becoming deprived in the near future. Such an approach assumes that political participation is integral to a stable democracy, as individuals must make their demands heard, voice their grievances, and hold politicians and governments accountable. If there is any doubt that the state is capable of managing the risks produced by and challenges of an economic crisis, it may lead to lower levels of political participation.

The extent to which the perception of one’s own risk of becoming deprived influences political participation has not yet been considered, although it is clear that deprivation has a strong subjective component (Galais and Lorenzini 2017; Grasso et al. 2017). This article attempts to close this gap in the literature and shows that the individual risk perception of becoming deprived increases the likelihood of participation. Accordingly, this article argues that individuals who *feel at risk* participate more as they may (still) see the possibility of bringing about change, while those who are already deprived seem to have lost hope of improvement and thus participate less.

Even though there are various studies on how economic performance affects levels of political participation, only a few deal with a time of real economic collapse (exceptions are Filetti and Janmaat 2018; Kern et al. 2015). In general, a crisis adds intense uncertainty to the lives of all individuals. Using data from the European Social Survey, this article examines the effect of this situation on political participation. The results show that, in non-crisis times, deterioration has a greater impact on personal perceptions of risk since improvement appears possible, increasing the likelihood of participation. However, in times of crisis, individuals seem to lose the hope of improvement and thus participate less. Political participation serves as a vehicle through which individuals communicate their interests and are able to put their public officials under pressure to take their interests into account. Thus, this finding leads to the alarming result that economic crises can further increase the political marginalisation of certain social groups.

This article proceeds as follows. The next section reviews the existing literature and develops subsequent hypotheses. After the description of data and methods used, the main analysis is undertaken in three steps. The investigation of the general relationship between individual risk perception and political participation is followed by an examination of the role of the affection of other group members and the context of the crisis. The article closes with a critical discussion of the main findings.

## Theory and hypotheses

Political participation is essential for the legitimacy of a democracy (Verba et al. 2002), and high levels of political participation are preferable. Two central questions in the research on political participation concern why people become politically active and what prevents people from participating.

Previous studies dealing with deprivation are often based on grievance theory, which offers insights into the relationship between different forms of deprivation and non-institutionalised political participation (Barnes et al. 1979; Gamson 1968). The reaction of an individual towards an objective circumstance heavily depends on his/her subjective way in which they judge their situation (Walker and Smith 2002). Hence the discrepancy between the value expectations and the value capabilities of an actor (Gurr 1970) is the core mechanism that leads to political mobilisation. Within this context, value expectations are conditions of life to which a person thinks s/he is rightfully entitled, and value capabilities are the conditions s/he considers her-/himself capable of getting and keeping (Gurr 1970; Lahusen and Kiess 2019). Relative deprivation can therefore be based on different causes, such as rising unemployment during the economic crisis (Tosun et al. 2017). It is important that relative deprivations largely depend on the frame of reference in which they are conceived (Runciman 1980). This reference frame also leads to the differentiation between individual and collective relative deprivation.

Individual relative deprivation means that a person is personally deprived in comparison to the generalised other. For example, one could be affected by the effects of the economic crisis and feel deprived in relation to others in general. Collective relative deprivation emerges when members of a group feel deprived because they feel they lack something to which they believe themselves legitimately entitled, compared to other groups. Smith and Ortiz (2002) have shown that feelings of collective deprivation promote political participation, whereas individual deprivation leads to personal reactions such as quitting one’s job or psychological depression. In this sense, individual deprivation is expected to lead to ‘exiting’ (Kriesi 2012), as previous research found that individual deprivation decreases the likelihood of participation.

Previous studies in this area make, even if they are based on other theoretical approaches,^1^ only examine actual deprivation (e.g. Grasso et al. 2019)—and none of them study individuals at risk of becoming deprived. There is no question that actual deprivation has an impact on political participation behaviour. However, existing research ignores the fact that the individual risk of becoming deprived increases, especially in the context of crises, and that it is only as a result of the crisis that entirely new groups are added to those potentially at risk. People who did not perceive any risk in the run-up to a crisis may experience a risk of becoming deprived soon, for example losing their job or expecting financial losses of another kind. This aspect, however, is especially important in today’s ‘risk society’ (Beck 1986), which is becoming increasingly preoccupied with notions of imposed risks, ranging from global warming to economic crises. Compared to other risks that individuals take voluntarily, understanding how individuals deal with these imposed risks, is the foundation of the relationship between individuals and their governance structures. An individual’s behaviour shows whether s/he believes that the state is able to manage the risk. Existing studies on voting for populist parties have already shown the important role played by the fear of, rather than the actual experience of, economic hardship. The underlying mechanisms identified here relates to the fear of job-loss due to automatization processes, which in turn increases support for right-wing populist parties, as they represent socially conservative values and want to turn back the clock to safer times (Kurer 2020). Therefore, this study contributes to the existing literature by showing how participation behaviour is influenced by the individual risk perception of becoming deprived.

But, what about those who are not yet exposed to direct deprivation, but who must fear that this will happen? Will those who are exposed to imminent threat also participate less? This article argues that this does not necessarily has to be the case. Research on emotions and politics has shown that, for example, anxiety and fear can prompt individuals to reconsider courses of action for dealing with the danger of becoming deprived (Brader and Marcus 2013; Galais and Lorenzini 2017; Jasper 2014). Miller and Krosnick (2004) argued that individuals change their political behaviour in the face of economic, social, or political change to avert the threat. Similarly, Kurer (2020) investigates the political consequences of occupational change and argues that employment trajectories and relative shifts in the societal hierarchy are central to understand voting behaviour. His results show that relative societal decline and concerns about one’s own position in the social hierarchy, instead of acute material hardship, drive support for right wing populist parties (Kurer 2020). This is due to the voters' desire that their perceived descent in the social hierarchy be addressed by the government.

Moreover, in the face of anticipated economic uncertainty, it could be viewed as an act of benevolence to refrain from taking steps to mitigate economic consequences. This does not mean that every individual act purely selfishly or constantly to maximize utility. It simply means that action is taken to avoid economic loss. This is exactly where the difference between potentially and actually marginalized individuals lies. The potentially deprived face the risk of losing membership in their social group and are afraid of social relegation, for example. At the same time, it could be argued from the civic voluntarism model (Brady et al. 1995; Kern et al. 2015) that one of the most important determinants is the availability of resources. From this theoretical view individuals who have enough time, money and civic skills will be politically active. Thus, based on resource mobilisation theory one could argue that the potentially affected still have the necessary resources for participation, i.e. are not already prevented from participating because of a lack of resources, and at the same time still have something to lose. Vice versa the expectation would be that in the context of an economic crisis resources are shrinking and thus political participation rates should decline (Kern et al. 2015).

This article thus argues that individuals who feel at risk but are not yet deprived see the possibility of averting the situation and may hope to do so through participation. Therefore, instead of a decrease in the likelihood to participate, this study puts forward the hypothesis that an increase in risk perception is accompanied by an increase in the likelihood to participate:

### H1

Individual risk perception increases the likelihood of political participation.

Furthermore, the relation between one’s own situation and the situation of others must be considered. Here, some authors speak of double relative deprivation, which suggests that the likelihood of participating increases when people feel that deprivation not only affects themselves but also other members of their social group (Foster and Matheson 1995; Grasso and Giugni 2016; Walsh 1981). This is not about simply adding deprivation, but about the interaction between individual and collective deprivation. Previous studies examined this as a cross-level-interaction with the expectation that in countries which were hit hard by the crisis the individuals who suffer become motivated to participate (Grasso and Giugni 2016). For the individuals affected in this way, ‘the personal become[s] political’ (Foster and Matheson 1995, p. 1168). The same could be expected of those who feel at risk of becoming deprived and also consider others in their country as being at risk. Following this line of reasoning, the second hypothesis considers this relationship:

### H2

Individual risk perception increases the likelihood of participating more strongly in a context where the mean risk perception on the collective level is high.

Finally, it is important to examine the extent to which the (country) context plays a role here. Do the expected effects differ according to whether it is a crisis or a non-crisis period? Research on institutionalized participation in particular is increasingly concerned with this interaction of personal material circumstances and contextual economic conditions. However, the focus is exclusively on acute economic deprivation and not on the risk perception of being deprived in the (near) future. The basic mechanism of action here is stated by Arceneaux (2003), who insists that it is not the economic conditions per se that are decisive, but who is attributed responsible for them. Those who suffer economically and hold the government responsible are more likely to vote than those who suffer economically and do not hold the government responsible (Arceneaux 2003). Subsequently, Incantalupo (2011) expects that individuals who lose their own jobs in times of high unemployment will perceive their situation as part of a larger social problem and that this will mobilize them to the ballot box. Finally, Aytaç et al. (2020) show that unemployment depresses participation, and it does so more powerfully under circumstances of low unemployment rates than under high unemployment rates. The finding is supported by a psychological argument similar to Arceneaux’s, in which job loss at low unemployment rates leads to self-reproach. If, on the other hand, one experiences job loss in times of high unemployment, the political level is blamed, and anger promotes participation at the ballot box. Furthermore, and in the context of the economic crisis of 2008, Kern et al. (2015) have shown that only unusually high levels of grievances have an effect on participation level. More recently, Filetti and Janmaat (2018) showed that the economic crisis did not change the propensity to engage but influenced the within-country distribution of active citizens. Further, from the literature on economic voting, it is known that individuals link their evaluations of the economic context to their votes (Hansford and Gomez 2015), and thus the perception of the national economy is partly decisive for participation (Hernández and Kriesi 2016).

The main argument here is that the risk of becoming deprived increases in times of crisis and the probability of the individual actually becoming economically deprived is significantly higher in times of crisis than in non-crisis times. An aspect that is also evident in the context of the most recent economic crisis, such as that related to the Covid-19 pandemic (Dhongde 2020). Subsequently, in the context of the present study, this aspect will find its way into the last hypothesis:

### H3

Individual risk perception increases the likelihood of participating more strongly in times of economic crisis than in non-crisis times.

In sum, this study expects the individually perceived risk of becoming deprived to increase political participation. Regarding context, the economic crisis can trigger this perceived personal risk. In the course of the economic deterioration, this leads to the expectation of a further increase in political participation within the crisis context.

## Data, variables and methodological approach

The analyses are based on the European Social Survey (European Social Survey 2008, 2016) for individual-level data, while country-level data were drawn from the World Bank. Waves 4 and 8 of the ESS contain the central independent variable for measuring individual risk perception. Wave 4 of the ESS was collected between August 2008 and March 2010 (European Social Survey 2018a), while wave 8 was collected between August 2016 and December 2017 (European Social Survey 2018b). The dataset includes 19 countries, namely Belgium (BE), Czech Republic (CZ), Estonia (EE), Finland (FI), France (FR), Germany (DE), Hungary (HU), Ireland (IE), Israel (IL), Lithuania (LT), Netherlands (NL), Norway (NO), Poland (PL), Portugal (PT), Slovenia (SI), Spain (ES), Sweden (SE), Switzerland (CH), and the United Kingdom (GB). These are the countries for which data are available for both waves. Data from the two waves were pooled for further analyses.

Research on political participation has evolved over recent decades. As the forms of individual political participation have changed, so has research (Weiss, 2020). The clear categorisation of the forms of political participation often seemed to be the goal. Since the abundance of forms of participation can lead to confusion or conceptual stretching, it is necessary to use clear definitions. Theocharis and van Deth (2018) offer a definition, whereby participation can be recognised as such if it is a voluntary activity that refers to people in their role as non-professionals and concerns government, politics or the state.

**Fig. 1:**
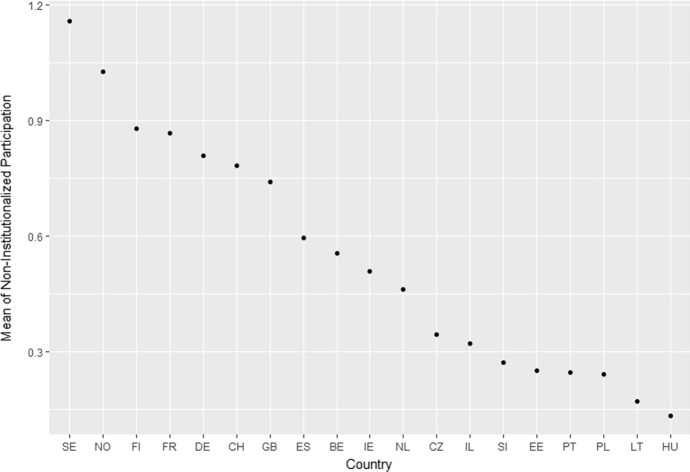
Mean of the dependent variable (non-institutionalized political participation) in the countries of the sample

The ESS contains several items for political participation. Following the multi-step classification according to Theocharis and van Deth (2018), the following variables can be used to measure the concept of non-institutionalised political participation: displaying campaign badges/stickers, signing a petition, taking part in lawful public demonstrations, and boycotting certain products. This study uses these items to construct the additive dependent variable of political participation. The dependent variable varies quite substantially across the 19 countries (see Fig. 1). Participation levels tend to be highest in the Scandinavian countries and lowest in the Southern and Eastern European countries.

To shed light on the role of individual risk perception on political participation behaviour, the central independent variable stems from the ESS. Respondents were asked how they assess their own risk of not having enough money for household necessities within the next 12 months. To measure the collective level, with the aim of testing the hypothesis on double deprivation, the next step was to calculate the mean personal risk perception by country. Previous studies have shown that comparisons with other countries play a less relevant role than the assessment of the national situation (Lahusen and Kiess 2019), and thus this variable is used to capture the group membership at the collective level. This country mean variable is then used together with the individual risk perception to calculate the cross-level interaction. The countries mean variable was calculated separately for each of the two waves of the ESS. The risk variables have been standardised for this purpose (Gelman and Hill 2006).

In order to examine the role of the crisis context, this study uses data from the World Bank (2020). In line with other studies (Kroknes et al. 2015), this article argues that to get a better understanding of how economic changes influence political participation behaviour, it is important not only to look at individuals’ perceptions of the economy, but also to look at the actual state of the economy. Thus, the macro-level variable for gauging problem pressure is change in unemployment.

Furthermore, a number of control variables are included. First, education is included (Berinsky and Lenz 2011; Marien et al. 2010), as previous studies found that better educated citizens are more likely to participate in forms of political behaviour (Hillygus 2005). Second, political interest is included, as high levels of political interest are linked to high levels of political participation. Third, gender forms part of the analysis, as women are expected to participate more often in non-institutionalised forms than men (Coffé and Bolzendahl 2010; Stolle and Hooghe 2011). Fourth, age is one of the control variables, as previous studies have shown that young adults are more likely to participate in non-institutionalised forms of political participation than adults (Fieldhouse et al. 2007; Quintelier 2007). Finally, the position in the labour force of each individual is included, as those who are still in education are expected to participate more than those who are employed, and those who are unemployed to participate less (Kern et al. 2015).

Since the data structure is hierarchical and individuals are nested within countries, this study uses multilevel modelling. More specifically, due to the composition of the dependent variable, linear multilevel models were employed. Further, random slope for the individual level were included since the assumption cannot be that the variation of the effect of the risk perception between countries can solely be related to macro-level predictors (Heisig and Schaeffer 2019). A description of all variables used within these models can be found in Table A.1 in the Online Appendix.

## Empirical findings

As this study utilises a hierarchical dataset, individuals (level 1) are clustered in countries (level 2). Furthermore, this study expects political participation to be shaped by both individual-level predictors and country-level predictors and by interactions between these variables. Table 1 presents the three models based on which the previously formulated hypotheses are to be tested. Hypothesis one is tested in the “M1” model. Hypothesis two is tested in the “M2” model, which introduces the interaction between country and personal mean risk perception. Finally, hypothesis three is tested in the “M3” model where the interaction between the change in unemployment and personal mean risk perception is included.^2^

**Table 1:** Non-institutionalized political participation

	M1		M2		M3	
	Estimates	CI	Estimates	CI	Estimates	CI
(Intercept)	− 0.051	− 0.175 to 0.073	− 0.475***	− 0.746 to − 0.205	− 0.035	− 0.160 to 0.089
Year (ESS wave 8 = 1)	0.042***	0.029–0.056	0.055***	0.037–0.073	0.024**	0.008–0.040
Risk of lacking financial resources	0.032***	0.016–0.047	0.149***	0.077–0.220	0.031***	0.016–0.046
Employment status^x^						
In education	0.043**	0.017–0.068	0.042**	0.017–0.067	0.042**	0.017–0.068
Unemployed	− 0.049***	− 0.078 to − 0.020	− 0.049***	− 0.078 to − 0.020	− 0.049***	− 0.078 to − 0.021
Inactive	− 0.094***	− 0.111 to − 0.077	− 0.094***	− 0.111 to − 0.077	− 0.094***	− 0.111 to − 0.077
Age	− 0.003***	− 0.003 to − 0.002	− 0.003***	− 0.003 to − 0.002	− 0.003***	− 0.003 to − 0.002
Gender (Male = 1)	− 0.106***	− 0.119 to − 0.094	− 0.107***	− 0.119 to − 0.094	− 0.106***	− 0.119 to − 0.094
Political interest	0.231***	0.224–0.238	0.231***	0.224–0.238	0.231***	0.224–0.238
Level of education^xx^						
Secondary education	0.178***	0.157–0.200	0.177***	0.155–0.199	0.177***	0.155–0.199
Tertiary education	0.395***	0.366–0.424	0.395***	0.367–0.424	0.394***	0.365–0.422
Countryriskmean			0.210***	0.097–0.323		
Interaction: countryriskmean × individualriskmean			− 0.058**	− 0.093 to − 0.023		
Change in unemployment					− 0.007***	− 0.011 to − 0.003
Interaction: change in unemployment × individualriskmean					0.001	− 0.000–0.003
*σ*^2^	0.66		0.66		0.66	
ICC	0.11		0.12		0.11	
Marginal *R*^2^/conditional *R*^2^	0.087/0.188		0.085/0.198		0.087/0.187	
Observations	69,883		69,883		69,883	
Number of countries	19		19		19	

*Source* Calculated from ESS data (waves 4 and 8) and World Bank data

^x^The reference category is in paid work

^xx^The reference category is primary education

*p < 0.05, **p < 0.01, ***p < 0.001

one asterisk is the lowest significance value

In a first step, this article examines the influence of individual risk perception on political participation behaviour. For this purpose, the individual-level model (see “M1”) includes both the central independent variable of personal risk to lack financial resources as well as the aforementioned control variables. This clearly shows that individual risk perception increases the likelihood of participation. Previous research in this area dealt with actual deprivation and often suggested that individual deprivation leads to ‘exiting’, thereby decreasing political participation (Kriesi 2012). However, this study shows that the opposite mechanism is the case when it comes to potential deprivation. As the personal risk perception of becoming economically deprived in the near future increases, so does the likelihood of participating. Thus, as long as individuals see the possibility of change or improvement, they participate more to avert the risk. Consequently, hypothesis one is confirmed.

With regard to the control variables and in line with previous research, it is evident that politically interested people are more active and that women are more active than men. In addition, a look at the educational level of the respondents shows that a higher level of education corresponds to a higher likelihood of participation. In relation to age, the likelihood to participate decreases with increasing age. Finally, in terms of employment status,^3^ those in education are more likely to participate than working people, while the unemployed and the inactive participate less.

**Fig. 2:**
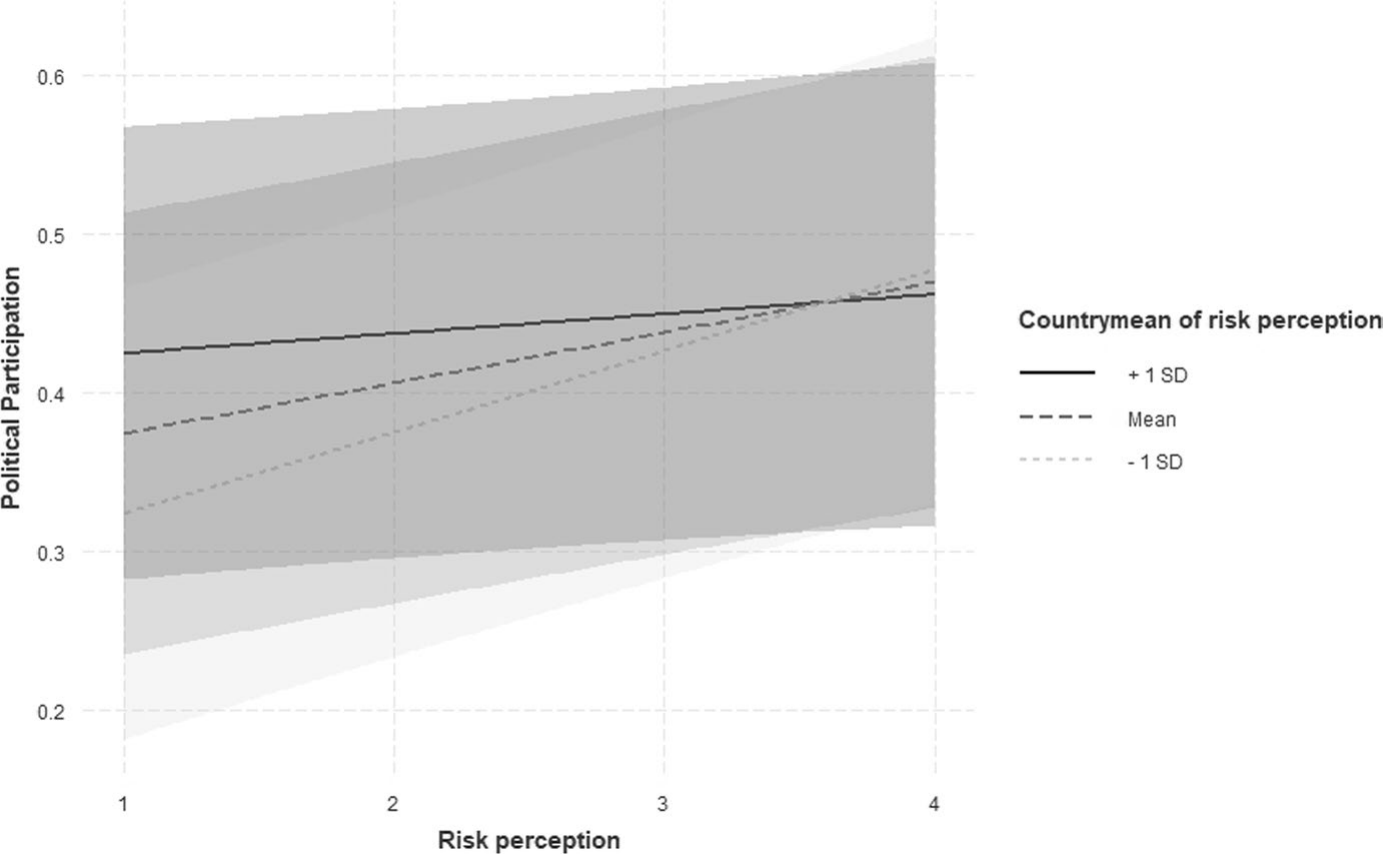
Cross-level interaction: Correlation between individual risk perception and political participation behavior interacted with the country mean of risk perception

The second step is to test the double deprivation theory. From a theoretical viewpoint, those who are themselves affected and who also perceive the group to which they belong as being affected should participate more. To measure group affectedness, a variable that contains the respective means of the risk variable in the individual countries was constructed. Here, the countries are understood as the respective group and the average risk perception of the respondents from the countries is determined. The result (see also ‘M2) show that the mean perception of risk in a country has a positive effect on non-institutionalised political participation, which means that as the average risk perception in a country increase, the probability of participation also increases. Further, a closer look at the cross-level interaction, which is also shown in Fig. 2, shows that this relationship is not the same for all individuals.

Instead, the cross-level interaction shows that the correlation between individual risk perception and participation behaviour becomes weaker as the average risk perception in the country increases. Thus, hypothesis two can only partially be confirmed. This supports the argument from previous studies that ‘the relative inertia of inequality gives rise to a sentiment that not much can be done about it’ (Filetti and Janmaat 2018, p. 344). Furthermore, this underlines that context plays a role (Ackermann 2017; Grasso and Giugni 2016) and that economically deteriorating situations in the country can reduce rates of participation. This is attributed to the fact that in the course of the ‘risk society’ a change in the relationship between the individual and the state has taken place (Curran 2013). Dealing with risks is characterised by increasing institutionalisation, which goes hand in hand with a political disempowerment of the individual. In this sense, political disengagement is a manifestation of the doubt that the state can deliver control or security in a context that poses a particular challenge to individuals.

The third and final step continues with the analysis of the specific context of the economic crisis. Do the effects found differ between crisis and non-crisis times or do they remain the same? To examine this aspect, a measure of the general situation, namely the change in unemployment, is added at the country level and the results for this are presented within the “M3” section.

**Fig. 3:**
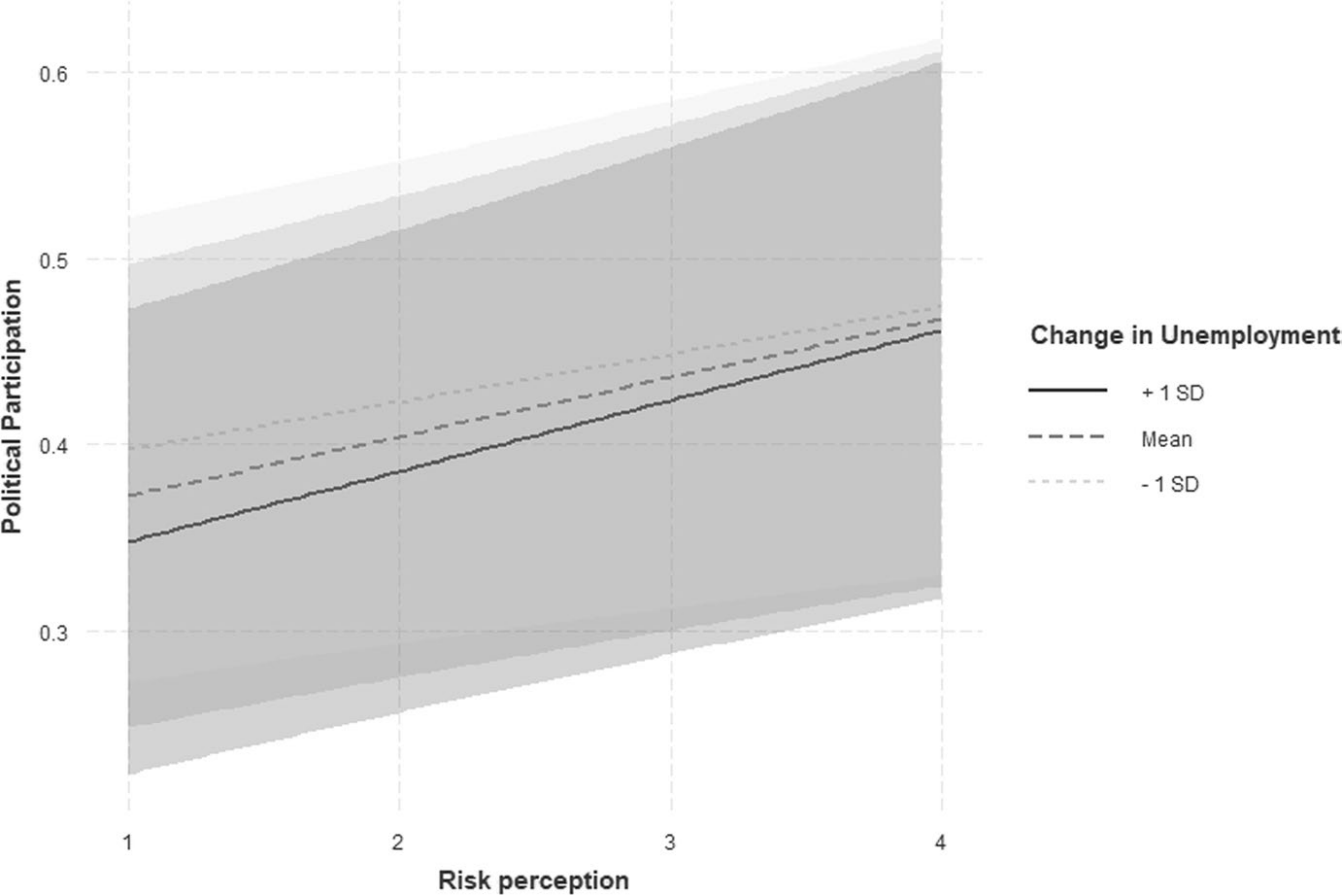
Cross-level interaction: Correlation between individual risk perception and political participation behavior interacted with countries change in unemployment

Regarding hypothesis three, the results show that the likelihood to participate decreases as change in unemployment increases. This stands in contrast to previous studies that expect an increase in the likelihood to participate for times of crisis and not for times of non-crisis (Kern et al. 2015). Conversely, the participation rate can thus be expected to rise again in non-crisis periods. Therefore, it cannot be assumed that in times of crisis an increase in deprivation is accompanied by an increase in political participation. Instead, it is again apparent that in times of crisis individuals lose hope that the state can bring about change and thus the necessary security for them. In non-crisis times, however, deterioration may have a greater impact on the perceptions of the individuals. Since in this context improvement should be possible it increases the likelihood of the participation of the individuals who demand this improvement. The corresponding cross-level interaction (see also Fig. 3) does not yield any significant result. Thus, in the future further analyses should investigate the possible relationship with regard to crisis and non-crisis periods in more detail.

In summary, the results underline the relevance of the context. The increasing likelihood to participate is especially the case when one perceives a risk yet also lives in a context in which improvement still seems possible. This is particularly evident with regard to the average risk perception in the country, i.e. the indicator of how people are doing or how they personally perceive their risk, irrespective of official unemployment statistics or indicators such as a country's economic performance. It is thus assumable that context shapes the connection between personal risk perception and individual participation behaviour (Ejrnæs 2017).

Nonetheless, there are limitations that must be considered. The survey data examined in this study highlights the need to take risk perceptions into account. However, only one form of risk perception could be considered due to data availability. Examining other risk perceptions, such as the risk of becoming unemployed,^4^ could further disentangle the relationship underlying the effect of potential deprivation on political participation. Additionally, mixed-methods approaches including qualitative interviews would provide additional insights into the patterns found in the analyses. For example, a rough demarcation of the social group had to be made within the scope of this study. For further in-depth studies of the double deprivation theory, it would be important to consider other forms of social group membership beyond that of the country.

Furthermore, the existing data situation results in an empirical strategy that does not allow for causal interpretations. Accordingly, there may be other interpretations of the mechanism of crisis on individual behaviour than the one presented here. For example, instead of the interpretation that individuals lose hope for improvement in times of crisis, one might consider whether individuals' priorities shift away from the political arena in bad economic times. Moreover, in this research design and with the available data, looking at the two points in time as well as the macro indicator of the change in unemployment, which was of central importance during the crisis period, can only provide initial interpretations in the direction of a crisis effect. Further research is needed here to examine in depth and further influencing factors of the crisis.

## Summary and discussion

Does the individual risk perception of becoming deprived influence political participation and, if so, does this depend on the economic context? The economic crisis that unfolded in 2008 provided the ideal framework for addressing this question empirically. Using data from the ESS and the World Bank, this study examined the role of the individual risk perception of becoming economically deprived on political participation behaviour.

The results showed that the likelihood of participation increases as the personal risk perception of becoming economically deprived in the near future increases. However, this initial positive effect for those who perceive their level of risk as being very high is diminished if the mean risk perception in the country is high and thus an economically weak situation prevails. Overall, the likelihood of participation particularly increases when one perceives a risk yet also lives in a context in which improvement still seems possible.

This analysis revealed that economic changes lead to new patterns of vulnerability, both on the individual and collective levels. If the context allows it, individuals who feel at risk participate more. The reason for this could be that these individuals (still) see the possibility of change, while those who are directly affected (e.g. by unemployment) seem to lose the hope of improvement and thus participate less. However, not only those directly affected participate less in times of economic downturn, but also those who perceive a high risk of becoming deprived for themselves. This result illustrates that instead of abstracts threats, such as the effects of climate change, risks in the immediate sphere of individuals are the main source of vulnerability in the current age (Lianos 2012). In this sense, both potential and direct economic deprivation translates into political marginalisation. This is particularly relevant when it comes to the effects of macro-economic conditions on democracy. Here, scholars have often expressed concerns regarding the vitality and stability of democracy (Inglehart and Welzel 2005). This involves the under representation of certain groups’ interests, which are now being reinforced in times of crisis. It turns out that not only one’s own affectedness, as shown by other studies, but also one’s own perception of the risk of becoming deprived, providing this takes place within a context that offers no hope of improvement, decreases the likelihood of participation. In this way, interests of these groups become further marginalised in the political process. Political marginalisation can not only be triggered by actual deprivation, but can start earlier—more precisely, with the very risk of becoming deprived. Nevertheless, the findings of this study are not only important with respect to the specific case of the economic crisis analysed here but suggest that future crises will further exacerbate the problem of political marginalisation of certain interests. The COVID-19 pandemic already made it clear that the economic crisis of 2008 would not be the last crisis. There is therefore an explicit need for a discussion on how individuals become politically empowered again, since no political governance can substitute for politically empowered citizens.

Related research from the field of populism additionally shows why it is important to look at status threat in the context of political participation. For example, Gidron and Hall (2017) or Mutz (2018) present status anxiety as a proximate factor inducing support for populism. Kurer (2020) shows in a similar way and with a view on occupational change, how the susceptibility to automation creates status anxiety, which then fuels support for right-wing populist parties. Thus, the political alienation of disadvantaged groups can encourage a vicious cycle of democratic erosion, by favouring policies (e.g., right-wing populist) that are disadvantageous to the disadvantaged, further increasing economic inequality and reducing the participation of the less well-off (Portos 2021).

Finally, this study provides an important starting point for further investigations. Further research on the influence of risk perceptions could be fruitful. The dependent variable in this study includes items for conventional forms of noninstitutional political participation, but excludes items for both institutional participation (voting, contacting elected officials) and unconventional forms of participation (violence, looting, rioting). Future research could determine whether deprivation has different effects depending on the area or form of participation. If panel data were to become available in the future that would allow a longitudinal view of individual life courses, it could also be investigated whether the assumption that willingness to participate increases when one considers oneself at risk and decreases as soon as one is affected is also evident for individual life courses and whether, for example, the role of trust in institutions has a demonstrable influence here. Furthermore, it would be important to investigate further the marginalisation of different groups (e.g. by class, similar to what Solt (2015) did for income groups). It would also be interesting to examine the perception of risk distinguished by the different institutional frameworks in which individuals live. This would entail the examination of what influence the respective welfare state regime has, for example, with regard to the question of whether the individual at risk of economic deprivation is more or less protected by the welfare state. Subsequently, new crises will provide the framework to study the robustness of the mechanisms identified in this study.

## Supplementary Information

Below is the link to the electronic supplementary material.

Supplementary file1 (DOCX 17 KB)

Supplementary file2 (DOCX 22 KB)

## Footnotes

1 These range from the focus on resources within the civic voluntarism model (Verba et al. 1995), the role of institutional settings within the framework of political opportunity structures (Grasso and Giugni 2016, Kitschelt 1986), to theories of collective action (Olson 1965; Opp 1990), to those which deal with the willingness of people to contribute to a public good (Ackermann 2017).

2 As a robustness check, all models presented in Table 1 were also done with a binary dependent variable. The binary variable takes the value 1 if one has exercised one or more of the four forms of non-institutionalised participation and the value 0 if one has not done any of them. The robustness check can be found in Table B.1 in the Online Appendix. The results confirm the correlations and directions presented in Table 1.

3 A look at the correlation between employment status as a possible indicator for actual deprivation and one's own risk perception (“Risk of lacking financial resources”) also shows that while those in work or education perceive a lower risk, those who are unemployed perceive a higher risk. The correlation between risk perception and employment status was calculated using Pearson’s correlation coefficient and showed the following results: In paid work − 0.075, In education − 0.032, Unemployed 0.20, Inactive 0.00. All correlations except for the Inactive present themselves as significant (*p* < 0.5).

4 This variable was included in the original questionnaire for the ESS rotation module ‘Welfare attitudes’, but was no longer asked in the 2016 wave. Therefore, this aspect could not be included in this analysis.
