# Supplementary material for: Disengaged or raising voices? An analysis of the relationship between individual risk perception and non-institutionalised political participation
Source: Acta Polit. 2023 May 13:1–19. Online ahead of print. doi: 10.1057/s41269-023-00301-x (PMC10182546; doi:10.1057/s41269-023-00301-x)
Supplement: Supplementary file 1 — Supplementary file1 (DOCX 17 KB) [file 41269_2023_301_MOESM1_ESM.docx]

**Appendix A**

Table A.1: Variable description

| Variable | Description | Range | Mean (SD) 2008 |
| --- | --- | --- | --- |
| *Dependent variable* |  |  |  |
| Non-institutionalised Political Participation | Additive index from participation variables:  ‘There are different ways of trying to improve things in [country] or help prevent things from going wrong. During the last 12 months, have you done any of the following? Have you…   - taken part in a lawful public demonstration? - boycotted certain products? - signed a petition? - worn or displayed a campaign badge/sticker?’ | 0-4 | 0.56 (0.90) |
| *Independent variables* |  |  |  |
| Risk of lacking financial resources | ‘And during the next 12 months, how likely is it that there will be some periods when you don’t have enough money to cover your household necessities?’ | 1-4 | 2.00 (0.89) |
| *Controls* |  |  |  |
| Age | Age of respondent | 15-123 | 48.65 (18.60) |
| Gender | 0= female, 1= male | 0-1 | 0.47 (0.50) |
| Political interest | ‘How interested would you say you are in politics?’ | 1-4 | 2.42 (0.91) |
| Level of education | Highest level of education  Primary education  Secondary education  Tertiary education | 1-3 | 2.01 (0.49) |
| Employment status | Current employment status  1 = In paid work; community or military service  2 = In education  3 = Unemployed (looking for a job and not looking for a job)  4 = Permanently sick or disabled; retired; housework, looking after children or other persons; other | 1-4 | 2.23 (1.38) |
| *Country-level variables* |  |  |  |
| Change in unemployment | The change in unemployment was calculated on the basis of total unemployment figures: Unemployment, total (% of total labour force) (modelled ILO estimate). Unemployment refers to the share of the labour force that is without work but available for and seeking employment. Since the shorter the time intervals selected for this purpose, the more random the values, the calculation of a value was always based not on the previous year but on two years before. | -14.16 – 18.24 | 0.59 (4.49) |
| Riskperception-Countrymeans | Mean of risk of lacking financial resources calculated by country. Calculation and assignment to the individual level data was done separately for the two ESS waves. | 1.43-3.05 | 2.00 (0.33) |
