# Supplementary material for: Disengaged or raising voices? An analysis of the relationship between individual risk perception and non-institutionalised political participation
Source: Acta Polit. 2023 May 13:1–19. Online ahead of print. doi: 10.1057/s41269-023-00301-x (PMC10182546; doi:10.1057/s41269-023-00301-x)
Supplement: Supplementary file 2 — Supplementary file2 (DOCX 22 KB) [file 41269_2023_301_MOESM2_ESM.docx]

Table B1. Robustness checks with binary dependent variable

|  | M1 | | M2 | | M3 | |
| --- | --- | --- | --- | --- | --- | --- |
|  | Estimates | CI | Estimates | CI | Estimates | CI |
| (Intercept) | 0.040 | -0.032 – 0.113 | -0.202 ^**^ | -0.352 – -0.051 | 0.049 | -0.024 – 0.122 |
| Year (ESS wave 8 =1) | 0.023 ^***^ | 0.016 – 0.030 | 0.030 ^***^ | 0.020 – 0.040 | 0.013 ^**^ | 0.004 – 0.021 |
|  |  |  |  |  |  |  |
| Risk of lacking financial resources | 0.012 ^**^ | 0.003 – 0.020 | 0.083 ^***^ | 0.043 – 0.122 | 0.011 ^**^ | 0.003 – 0.019 |
|  |  |  |  |  |  |  |
| Employment status^x^ |  |  |  |  |  |  |
| In education | 0.018 ^**^ | 0.005 – 0.032 | 0.018 ^**^ | 0.004 – 0.031 | 0.018 ^**^ | 0.005 – 0.031 |
| Unemployed | -0.027 ^***^ | -0.042 – -0.011 | -0.026 ^***^ | -0.042 – -0.011 | -0.027 ^***^ | -0.042 – -0.012 |
| Inactive | -0.043 ^***^ | -0.052 – -0.034 | -0.043 ^***^ | -0.052 – -0.034 | -0.043 ^***^ | -0.051 – -0.034 |
| Age | -0.001 ^***^ | -0.002 – -0.001 | -0.001 ^***^ | -0.002 – -0.001 | -0.001 ^***^ | -0.002 – -0.001 |
| Gender (Male=1) | -0.046 ^***^ | -0.053 – -0.040 | -0.046 ^***^ | -0.053 – -0.040 | -0.046 ^***^ | -0.053 – -0.040 |
| Political interest | 0.113 ^***^ | 0.109 – 0.116 | 0.113 ^***^ | 0.109 – 0.116 | 0.113 ^***^ | 0.109 – 0.116 |
| Level of education^xx^ |  |  |  |  |  |  |
| Secondary education | 0.110 ^***^ | 0.098 – 0.121 | 0.109 ^***^ | 0.097 – 0.120 | 0.109 ^***^ | 0.097 – 0.120 |
| Tertiary Education | 0.211 ^***^ | 0.196 – 0.226 | 0.211 ^***^ | 0.196 – 0.226 | 0.210 ^***^ | 0.195 – 0.225 |
| Countryriskmean |  |  | 0.120 ^***^ | 0.058 – 0.181 |  |  |
| Interaction: Countryriskmean x Individualriskmean |  |  | -0.035 ^***^ | -0.055 – -0.016 |  |  |
| Change in Unemployment |  |  |  |  | -0.004 ^***^ | -0.006 – -0.002 |
| Interaction: Change in Unemployment x Individualriskmean |  |  |  |  | 0.001 | -0.000 – 0.001 |
| σ^2^ | 0.18 | | 0.18 | | 0.18 | |
| ICC | 0.12 | | 0.14 | | 0.12 | |
| Marginal R^2^/ Conditional R^2^ | 0.077 / 0.191 | | 0.075 / 0.202 | | 0.078 / 0.191 | |
| Observations | 70059 | | 70059 | | 70059 | |
| Number of countries | 19 | | 19 | | 19 | |

Note: x=The reference category is in paid work. xx= The reference category is primary education.

Source: Calculated from ESS data (waves 4 and 8) and World Bank data.

^*^ *p* < 0.05, ^**^ *p* < 0.01, ^***^ *p* < 0.001
